# Dental Transplantation and Replantation

**Published:** 1889-02

**Authors:** H. A. Smith

**Affiliations:** Cincinnati, Ohio


### THE DENTAL REGISTER.

Vol. XLIII.] FEBRUARY, 1889. [No. 2.

### COMMUNICATIONS.

### Dental Transplantation and Replantation.

BY H. A. SMITH, D.D.S., CINCINNATI, OHIO.

[Read Before the Ohio State Dental Society, Cincinnati, October, 17, 1888.]

In looking up the history of Dental Transplantation and Replantation we find that they have quite a respectable antiquity and were probably practiced as early as any operation in dental surgery. Ambrose Pare, who died near the close of the sixteenth century, mentions in his work cases of transplantation of natural teeth ; he also describes in detail the operation of replantation.

As early as 1686 there is recorded a definite opinion as to the practicability of the replantation operation ; thus Thos. Berdmore in that year says, "Replantation, I regard as easily done though of doubtful utility." "The practice of replantation," he continues, "is not only pernicious, ineffectual, and dangerous in general, but immoderately expensive." His methods of preparing a tooth for replantation did not differ materially from those now in use. He recommended that the root canals be filled with gold or lead.

M. Patence, of London, in 1774 calls attention to the "practice of extracting the teeth of one person and replacing them in the sockets of another." He says, "It must certainly be highly offensive to the Almighty, for He never gave them for that purpose, neither are they their own to dispose of." He also records a case in which syphilis was transmitted by transplantation. Speaking of the preparation for transplantation of a dried tooth taken from a skull, he says, "Lay it in water for three months to soak that it may open the pores, then put in hot water for three hours." From this it will be seen that even at that date quite an effective method of sterilizing a tooth was in practice.

Mr. Bell in his edition of Hunter's "Treatise on the Teeth," erroneously attributes the origin of the practice of transplantation to his author. Hunter, it appears, had frequently transplanted teeth and describing what he considered the manner of attachment, says: "The success of the operation is founded on a disposition in all living substances to unite when brought into contact with one another, although they be of different kinds of structure." He demonstrated this theory by transplanting a freshly extracted tooth in a cock's comb. After some months the cock was killed and an examination made of the tooth *in situ*. He found the vessels of the tooth well injected and the external surface of the tooth firmly adherent to the comb—a union very similar to the natural one in the gum. This would indicate that the modern theory of revivification of the tooth is a revival of and not an advance on the old Hunterian theory.

Surgical operations are governed somewhat by fashion, and since transplantation was first practiced—now certainly more than 200 years—it has had the characteristic periods of favor and disfavor. In our own experience we have seen its rise and fall a number of times. Transplantation and the kindred operation of replantation are justifiable and valuable in certain emergencies, and since we now have at our command ready methods of antisepsis, together with a better understanding of the physiological and pathological conditions attending the operations, no trained dentist need hesitate to perform them in suitable cases. But there are now so many excellent methods of replacing lost teeth with artificial substitutes, that there is not that necessity of resorting to the plantation operation that there once was.

The operation of restoring to their sockets teeth that were either accidentally displaced or removed for cause has been practiced several centuries. Doubtless parents have always been instinctively prompted to put back in the sockets their children's teeth when displaced by accident. All of us have had such cases brought to our notice, and when we consider that the pulps in these teeth had not been removed it is remarkable how enduring and comfortable has been their retention. It is still a question whether or not the pulps of teeth that have been immediately returned to their sockets in young and healthy subjects retain their vitality. Divided nerve tissues are prone to unite under favorable circumstances, but it would seem that the conditions brought about by violently severing the pulp at the apex, are most unfavorable for a reunion. The seemingly healthy state of these restored teeth is often misleading, and in a majority of such cases the pulp will sooner or later take on the putrefactive stage.

Perhaps no one reports a larger percentage of success in replantation than does Magitot, of Paris. He has given us a record of 63 cases out of which only five had failed.

Dr. G. R. Thomas, of Detroit, reports nearly 500 cases of replantation, and says that many of them are doing good service. Yet he expresses doubt as to the permanent benefits to be derived from the operation.

Dr. G. W. Weld in the *American System of Dentistry* reports 80 cases of replantation. After carefully watching these cases he concludes that replanted teeth readily attach themselves and become firm in their sockets; that they remain useful for varying lengths of time, dependent upon local and systemic conditions; that a full vital relation rarely occurs; that death of the peridental membrane, cementum and dentine speedily follows; that, owing to the death of the tissues and consequent breaking up of the attachment, such teeth are lost by gradual loosening as the result of acute inflammation set up about them; that, in view of possible danger and the certainty of ultimate failure, the practice of replantation, except in cases in which teeth are displaced by accident, is not justifiable.

I lately received from Dr. A. G. Weber, of Havana, Cuba, an interesting report of 14 cases of transplantation and 12 cases of replantation. The history of these cases is the usual one (when the operations are made by careful dentists). For a time varying from a few months to five or seven years these teeth were retained with comparative comfort to the patients. Case XV

well illustrates the power of riches: May 25, 1880, transplanted a superior central incisor which was purchased by the patient at the price of $102, Spanish gold. It had brilliant success until February 16, 1885, when it had to be extracted on account of absorption of the root.

---

DISCUSSION.

*Dr. Berry*—History repeats itself. Solomon said so three thousand years ago, and the statement is as true now as it was then.

The first thing on the announcement of this meeting is 1788, and opposite is 1888. It required some study to understand the meaning of these figures. But it is evident that transplanting teeth is the only subject in the programme to which they can have reference. About 1788 transplanting teeth was practiced by many dentists in Europe and in this country. Among them was the celebrated John Hunter, who, of all the dental authors of distinction, is the only one who recommended the transplantation of teeth.

In the winter of 1785 and 1786, Mr. Lemayeur, in Philadelphia, transplanted one hundred and seventy teeth, as he told Dr. Gardette. Dr. Gardette says, that "of all these transplanted teeth not one succeeded," although "some became firm and lasted, more or less, for one or two years," and that he extracted at least fifty of them. The want of success and danger of communicating disease brought this practice into disrepute. Dr. Gardette relates two cases of Dr. Lemayeur's patients, who had syphilis communicated by the transplanted teeth, from which they died.

Now, a century later, comes a revival of the practice of transplanting teeth, by Dr. Younger's process, differing somewhat from that of the former time, but equally dangerous as to the transmission of disease.

Why are not women carried through child-birth now as was done some years ago by Sir James Simpson, and other eminent physicians, by the use of chloroform? Simply from the danger attending it. But the proportion of fatal cases from the use of chloroform was greatly less than from transplanted teeth.

From the period of the revival of transplanting teeth by Dr. Younger, I have spent considerable time studying to devise means to insert teeth on Dr. Younger's plan without danger of transmitting disease. Probably porcelain teeth, with the parts to be in sockets ground and closely fitted, would be retained, and if the inserted portion was wood, it would undoubtedly be held in place.

*Dr. Fletcher*—In explanation of Dr. Smith's statement, that I prefer dry teeth for implanting in comparison with those having been preserved in a fluid, I would say, the dry periosteum is preferable in my estimation, for the reason that the cells composing the structure of that membrane, would probably imbibe any fluid in which they were immersed any length of time, and this I think would be fatal to their life, supposing it to be true that they can revive when the tooth is implanted. Whereas a dry cell when placed in its natural environments could imbibe only its natural fluids, and it seems probable that these cells could revive only when furnished with these life-giving elements, and these at their proper temperature, all of which are found in the blood.

We know it to be a fact that some vegetable cells lie dormant for an indefinite number of years and then revive, producing their kind when surrounded with the proper elements and temperature, but the main requisition of such a dormant condition is that the cells be kept perfectly dry. To immerse it in any kind of fluid for even a few hours would destroy its power of revivification.

Now, since cell life—from our present knowledge—seems much the same whether animal or vegetable, does it not seem probable that we do have animal cells which may have this same power of dormant life; if so, they would most likely be controlled by much the same conditions. If it be a fact that dried teeth and those having been preserved in a fluid, could both become equally firm and useful as an implanted tooth, it would to me be a strong proof that the peridental membrane did not revive, but acts only as a protection to the root.

As to antisepsis, I believe all the antisepsis these membranes need can be given at the time of the operation by immersing the tooth for a few moments in a one to one thousand solution of bichloride of mercury, and this while the operation is in progress.

There is one point in the manipulation of these cases I did not mention, which seems to me to be of importance, and that is, while drilling the socket, or after its completion, the chips should not be washed out, but left in to form nuclei, for the growth of new bone-cells. Each chip of bone, however small, must contain either a whole or part of a bone corpuscle, which corpuscle I believe will become a nucleus for new bone material, in the same manner that very minute particles of the skin do in skin-grafting.

Now, why may not these small particles of bone perform the same office in this operation that the small particles of skin do in skin-grafting, and if they do we certainly shorten the time of recovery, and probably secure the chances of success. As to the necessity of washing the new socket with an antiseptic fluid it would seem ample to have the hands and instruments thoroughly sterilized by an antiseptic fluid before beginning the operation, and the tooth treated in the same manner; certainly no septic germs other than those which may be in the blood of the patient could be found in the socket.

In a paper on implantation read before the Mississippi Valley Dental Association last March, I gave some evidence of the revivification of the peridental membrane. Nevertheless, I believe the bone chips left in the socket in these cases, to be of as much advantage in the repair of lost tissue and their rapid recovery, as the presence there of the peridental membrane on the tooth. However, at our present state of knowledge, I believe it the safe plan to implant only teeth whose roots are covered with membrane.

*Dr. Arnold*—I have had some little experience in replantation. I have one case that I would like to speak of in particular. It is now of ten months' standing. After my return from the Congress at Washington, where I saw some of Dr. Younger's clinics, some enthusiastic gentleman came to my office one day to have a tooth reconstructed—a lateral superior incisor. It struck me immediately that there was a good chance for implantation if he would submit to the operation; I thought favorably of it. I

spoke of it in this way and said that as soon as I could get a suitable tooth I would perform the operation; and he said "I have the tooth; I lost it ten years ago; I have it somewhere at home; I will make search for it and bring it to you." He brought the tooth and I placed it in an antiseptic solution of mercury and finally inserted it. The tooth was implanted; ten months have elapsed, and to-day the tooth is apparently as good as any other tooth he has. It was the only tooth he had ever lost. To-day that tooth is in good condition, and free from all irritation, my conclusions are that there is an ancyhlosis. For this reason I prefer a dry tooth and I shall continue to make operations that way. There is one thing about a dry tooth, one that is perfectly dry becomes friable and of course the preparing of such a tooth must be done very carefully. According to my theory I cover the tooth at the root or periosteum with the best of mucilage, so I can protect it while handling, then I dry the tooth and work very slowly. As to the age of the tooth I think it would be better to have a tooth from a young person.

In the replantation of teeth my experience has taught me that they become solid in about three months. The color of the tooth while not very much different at the time of implantation, a slight difference can be seen and the chances are ultimately perhaps that it will become thoroughly imbued with the pigment that we find in the normal condition. Now I was very careful in operating, at least I used my best efforts to that end. I think the proper requirements—indeed the necessary requirements—in all cases of that kind, should be to begin with a good systemic treatment of your patient. The other requirement is perhaps the most important after all—antiseptics. I need not only to immerse the tooth in a solution of one and a half per cent. of mercury, and syringe the socket with the same solution, because I regard it as highly important that the socket as well as the tooth be placed in an antiseptic condition. I am a great believer in antiseptics. I think that modern surgery is a success as practiced to-day in all the very elaborate surgical operations, because of the use of antiseptics. While I am convinced that the antiseptic practice very much lessens the risk of any operation, I should be very particular to ascertain when a case presents itself, to discover if possible whether there be any specific trouble in the patient. In this instance the tooth came from the same patient, and after ten months there is not an iota of any degeneration. On the contrary there is every reason to believe that it is improving all the time. Now, there is union; if there were not union, I do not think it would support it more than it would a peg.

*Dr. Wright*—We have, we may say, two sorts of knowledge in our profession, one is the clinical and the other is the scientific. On this question of implantation of teeth we are in the infancy of practice, and I think it would be well to experiment in order that we can be in accord in regard to the operations and thus acquire a clinical knowledge. So far as our scientific knowledge goes, we are still at sea, and we are possibly not correct at all. Still operations can be performed and successfully performed without this scientific knowledge. Surgeons have bandaged fractures and the bones have united so as to enable the patients to use their limbs perfectly well.

One point referred to by Dr. Fletcher, in regard to leaving chips in the socket. It seems to me we are all off in regard to that. If our modern ideas or theories are correct in regard to the formation of the bone, I suppose that these chips do not serve as nuclei in the formation of bone or cartilage.

Now as far as the clinical knowledge is concerned, it seems to me that we are doing splendidly, and I think Dr. Fletcher, who has had the courage to go into this first, and some others, should have their names honored.

*Dr. Bell*—I have had very little experience in the replantation of teeth myself, and not any in implantation. I was present at the Northern Ohio Society a few years ago and asked Dr. Thomas about transplanting teeth, and he said he was at one time very enthusiastic in regard to the matter, that he had treated altogether 500 cases, and that two years afterwards he had seen between two and three hundred cases and almost all were failures. He also said that of the number treated, practically all would be failures.

*Dr. Butler*—For many years in surgery, especially in cases of fractures, various means have been adopted whereby these fractures may be repaired or assisted in the reparation, and some recent experiments have been made by implanting bones into these fractures for the purpose of assisting nature in forming the union. Now if that be a practical thing, I cannot see any inconsistency in leaving these chips that are spoken of by Dr. Fletcher.

